# Editor's Report

**Published:** 1948

**Authors:** 


### Editor's Report

IN the Editorial Report last year it was pointed out to the Society that the increased cost of printing would add considerably to the cost of producing the *Journal.* This year it is possible to say what the charges for the normal four issues have been. The details are as follows :—

| | | | £ | s. | d. | No. of Pages |
|---|---|---|---|---|---|---|
| No. | 227. | Autumn, 1946 ... ... | 67 | 19 | 0 | 89—123 |
| ,, | 228. | Winter, 1946 ... ... | 71 | 16 | 1 | 125—161 |
| ,, | 229. | Spring, 1947 ... ... | 90 | 5 | 4 | 1—30 |
| ,, | 230. | Summer, 1947 ... ... | 57 | 13 | 1 | 31—60 |
| | | | £287 | 13 | 6 | |

In the spring number four original papers were published as well as the list of subscribers and the index, but the number of pages was not increased.

The setting up of the Subscribers' List was the main extra expense.

It has been difficult to compare the cost of producing the *Journal* with that during the few years before the war because the whole of the *Journal* account books have unfortunately been lost.

I have, however, found a statement in the Editorial Secretary's Report of 1898 which is interesting. In that year the *Journal* receipts were £224 16s. 11d., and £206 17s. 10d. was spent, leaving a balance of £17 19s. 1d.

From the Secretary's Report for 1898 it appears that Subscriptions to the Society amounted to £184 16s. 0d., the largest amount ever received.

The cost of production of the *Journal* during the war years was as follows :—

| | £ | s. | d. |
|---|---|---|---|
| Autumn, 1939 / Summer, 1940 ... ... ... | 181 | 2 | 3 |
| Winter, 1940 / Spring, 1941 ... ... ... | 123 | 12 | 3 |
| Autumn, 1941 / Spring, 1942 ... ... ... | 152 | .14 | 5 |
| 1942 ... ... ... ... ... | 48 | 0 | 9 |
| 1943 ... ... ... ... ... | 90 | 3 | 7 |
| 1944 ... ... ... ... ... | 91 | 16 | 9 |
| 1945 ... ... ... ... ... | 171 | 16 | 11 |
| Spring, 1946 / Summer, 1946 ... ... ... | 171 | 10 | 3 |
| | £1030 | 17 | 2 |

From these figures it will be seen that the annual cost of the *Journal* to the Society did not increase during the war years.

